# Similarity in Food Cleaning Techniques within Matrilines in Wild Vervet Monkeys

**DOI:** 10.1371/journal.pone.0035694

**Published:** 2012-04-25

**Authors:** Erica van de Waal, Michael Krützen, Josephine Hula, Jérôme Goudet, Redouan Bshary

**Affiliations:** 1 Institute of Biology, University of Neuchâtel, Neuchâtel, Switzerland; 2 Applied Behavioural Ecology and Ecosystem Research Unit, University of South Africa, Florida, South Africa; 3 School of Psychology, University of St Andrews, St Andrews, United Kingdom; 4 Evolutionary Genetics Group, Anthropological Institute and Museum, University of Zürich, Zürich, Switzerland; 5 Department of Ecology and Evolution, University of Lausanne, Lausanne, Switzerland; CNR, Italy

## Abstract

Social learning and the formation of traditions rely on the ability and willingness to copy one another. A central question is under which conditions individuals adapt behaviour to social influences. Here, we demonstrate that similarities in food processing techniques emerge on the level of matrilines (mother – offspring) but not on the group level in an experiment on six groups of wild vervet monkeys that involved grapes covered with sand. Monkeys regularly ate unclean grapes but also used four cleaning techniques more similarly within matrilines: rubbing in hands, rubbing on substrate, open with mouth, and open with hands. Individual cleaning techniques evolved over time as they converged within matrilines, stabilised at the end and remained stable in a follow-up session more than one year later. The similarity within matrilines persisted when we analyzed only foraging events of individuals in the absence of other matriline members and matriline members used more similar methods than adult full sisters. Thus, momentary conversion or purely genetic causation are unlikely explanations, favouring social learning as mechanism for within matriline similarities. The restriction of traditions to matriline membership rather than to the group level may restrict the development of culture in monkeys relative to apes or humans.

## Introduction

Human culture is studied with great interest and its comparison with other animals has become a broad research topic [1]. Culture and traditions are based on social learning, and there is a large body of literature covering the subject [2]. Typically, experiments about the underlying copying mechanism in animals have been conducted in the lab [3]–[6]; some studies have succeeded in testing experimentally also some wild animals [7]–[12]. However most of the results on social learning in the wild are either documentation of spread of new techniques or behavioural differences between populations [13]–[15].

Not only is the existence of social learning studied, but researchers are also more broadly interested in the rules of social learning under various contexts (e.g. when, what, who is copied). The usefulness of social learning is dependent on the conditions and on the presence of suitable models and therefore may not always be the most beneficial strategy for an individual [16]–[21]. Social learning can arise from conformity (sensu [16]) which means copying the majority. Alternatively one could use a class of individuals as models like members of the philopatric sex, or specific individuals (like older or dominant ones). The degree of uniformity of behaviour expressed in a group will depend on the number of suitable role models. If all individuals copy the alpha individual behaviour will be more uniform than if all mothers are copied by their respective matriline members. As a consequence, social learning rules will have a major influence on the scale of traditions. Yeaman *et al*. [22] modelled social learning rules in combination with migration patterns and found that the interaction produces conditions in which traditions may form on the population level and conditions under which traditions may form on the group level or within even smaller units. Observations on foraging technique development suggest that in several wild primate species, mothers are central for technique acquisition [23]–[27]. On the other hand, it is often assumed that differences in traditions are expressed on the population level [14]–[15]. Explicit experiments on a number of sympatric groups may help to elucidate social learning rules.

Here we provide an experimental approach aimed at identifying units for social information transfer in wild vervet monkeys. We provided six groups with grapes covered in sand and noted who cleaned the food before eating and how. The experiment simulated the food cleaning context that provided the first example of a tradition in wild primates, the sweet potato washing Japanese macaques [28]. We conducted 15 sessions (minimum time interval of ten days between sessions). After a minimum of a year without testing, we conducted a 16^th^ session. For all our analyses of units of social information transfer, we distinguished the following: matrilines (mother and offspring), females only as the philopatric members of the group, and the entire group. Matriline membership was initially assessed based on behavioural observations but genetic analyses of 74 individuals were available to confirm and identify relatedness coefficients between adult females.

Our hypotheses were based on previous research on the same six vervet groups. Most importantly, vervets pay more attention to dominant females than to dominant males in a social learning task and are hence more likely to learn from the females [11]. In the current experiment, high ranking individuals could potentially serve as models for lower ranking group members as the food source was clumped and hence dominants ate first while subordinates had to wait and thus observing the techniques used by dominants. Therefore, if dominant females are key social models as predicted by de Waal [18], we expected to find similarities in cleaning techniques on the group level. If females are generally key social models for members of their own matriline, we predicted similarities in cleaning techniques on the matrilineal level. As foraging techniques could potentially change over the course of the experiment we also tested how stable the techniques used by individuals were over the course of the 15 sessions, and then again after one year. Due to individuals foraging sequentially, we also investigated which individuals fed simultaneously and whether frequent co-feeding correlated with similarity in cleaning techniques during co-feeding events and/or also in situations when these partners fed independently. Finally, we contrasted the social learning hypotheses with a more genetic mechanism underlying cleaning methods. If there was a strong genetic basis we would predict that full adult sisters – now having their independent matrilines – use similar techniques with the same probability as mothers and their offspring. We will discuss the implications of our results for the establishment of traditions in vervet monkeys and consider potential underlying mechanisms.

## Materials and Methods

### Ethical Statement

Our experiments were approved by ABERRU boards of UNISA as well as Park Boards of the Mpumalanga Province, South Africa. Our experimental set-up involved feeding in enough quantities for all group members to access.

### Study site and population

Experiments were conducted between 2006 and 2009 on six neighbouring groups of habituated wild vervet monkeys (*Chlorocebus aethiops*) at Loskop Dam Nature Reserve, Mpumalanga Province, South Africa. The reserve covers 25'000 ha and was created in 1948. During our study, the vervet monkeys lived in stable family groups which varied from ten to 27 individuals. Groups are typically composed of an alpha male, a few subordinate males and several matrilines, i. e. females and their offspring. Females remain in their natal group all their life, while males migrate to another group when they are sexually mature, usually around 4 years of age. Vervets are described as opportunistic omnivores and readily eat human food if available. Our six study groups – Picnic, Nooitgedacht, Blesbokvlakte, Donga, Bay and Fishing Camp (named after sites on the Park map) – live in contiguous home ranges along a tourist road that allows easy access to each group. Group compositions are summarized in Table 1. All groups had been exposed to the presence of human researchers for at least six months before they were tested. Two of the six groups were in regular contact with tourists; one at a picnic spot (‘Picnic group’) and the other one at a fishing camp (‘Fishing camp group’). The ‘Picnic group’ and the ‘Donga group’ had been used for experiments before [29], and artificial fruit experiments [11], [12], [30] were conducted in parallel on all six groups.

**Table 1:** The composition of the study groups.

Group	Adult male	Adult female	Juvenile	Total
Bay	4	5	12	21
Picnic	3	3	10	16
Blesbokvlakte	2	3	8	13
Donga	4	6	5	15
Nooitgedacht	3	4	10	17
Fishing Camp	3	4	15	22

Males are scored as adults once they migrated, while females are scored as adults once they have given birth. Group members that did not fulfill these criteria were scored as juveniles.

All individuals were identified by their faces, and a recognition file with portrait pictures and specific individual features (scars, etc) was constructed for each group. Monkeys were named with letter codes. Individuals belonging to the same matriline share the same first letter. We coded females using the first three letters and males using the first two letters of their names. Matriline membership assignment was initially based on behavioural data: mothers nursing infants and adult females frequently being close and tolerant of juveniles in feeding and resting contexts were taken as evidence for matriline membership. Genetic data based on DNA extraction from faecal samples conformed to our classifications in all available 42 infants/juveniles – behaviourally assigned mother pairs.

### Food cleaning experiment

We designed our own experiment based on the sweet potato washing observations among Japanese macaques [28] that had documented food cleaning traditions in primates for the first time. We provided the vervets with a plastic box (34×14×12 cm) containing grapes covered with sand (100 g of sand for 2 kg of grapes) in quantities that even subordinates could eventually access the food, typically after dominant individuals had finished eating. The box was fixed on the ground using a rope and tent pegs. We first conducted a control test offering clean grapes, to habituate the monkeys to eating grapes. Then we conducted ten sessions with grapes covered in sand. Every minute we noted who was eating at the box and who was within a diameter of ten meters. We used the focal sampling method, aiming to observe how each individual processed ten grapes per session. In addition, all sessions were video-taped so that we could complete data sets on individuals for which we had not obtained ten observations during a session. Focals started only after the individual had started eating for at least 60 s in order to avoid that being still unsettled may affect grape handling. We identified four different cleaning techniques:, rubbing the grape in the hands, rubbing the grapes on substrate (ground, branches, stones, the plastic box), opening the grape with the teeth and eating the inside without the peel, opening the grape with the hands and eating the inside without the peel; finally some monkeys ate the grapes directly with the sand, called ‘no cleaning’. After the first ten sessions we added a second plastic box of the same size with water and continued data collection for another five sessions. As none of the monkeys ever used the water to clean grapes we could analyze all 15 sessions in one data file. A total of 98 individuals that participated in at least 10 sessions were included in our analyses. With the help of linear mixed effects models we could compare the relative importance of sex, age, kinship, group affiliation, for the different cleaning techniques used, as well as the evolution of these techniques throughout the 15 sessions. A minimum of one year after session 15, we conducted another experiment in all six groups and analyzed it in the same way as all first 15 sessions.

Initially the monkeys would only dare to eat grapes while facing us but eventually became more habituated to our presence and to the experimental setup and started turning their back to us and thereby obscuring their own actions as well as actions of others. We therefore fixed a Plexiglas plate (60×30×0.4 cm) in front of the box so that the monkeys had to face us.

### Genetic analyses

We extracted DNA from fecal vervet samples using the QIAamp DNA Stool Mini Kit (QIAGEN), following the manufacturer's protocol with one modification: samples were allowed to incubate for a minimum of 30 minutes before elution. We quantified DNA through real-time quantitative polymerase chain reaction (rtPCR) as in Morin et al [31]. This rtPCR assay allows determination of the number of positive PCR replicates per extract necessary to obtain a 99% confidence level that a homozygous genotype is correct. For a heterozygous genotype, our criterion was each that of the two alleles needed to be observed at least twice in independent PCRs. Ten randomly chosen individuals were extracted and genotyped independently for a second time in order to calculate our genotyping error rate.

PCR amplifications for 13 human-derived microsatellite loci [32], [33] were performed as multiplex reactions in an 10 µL volume containing 1 µL DNA, 5 µL Multiplex Master Mix (QIAGEN), 1 µL primer mix (diluted 1∶5), and 3 µL ddH_2_O. Amplification conditions were: initial denaturation at 95°C for 15 minutes, followed by 40 cycles of 94°C for 30 s, 58°C for 90 s, 72°C for 1 min, and a final extension at 60°C for 30 mins. We performed capillary electrophoresis on the 3730xl DNA Analyzer (Applied Biosystems). Products were analysed using GeneMapper v4.0 (Applied Biosystems). We used Genepop v. 3.0 [34] to calculate deviation from Hardy Weinberg equilibrium and linkage disequilibrium. We checked for allelic dropout and null alleles using Microchecker 2.2.3 [35].

Pairwise relatedness estimates for 74 monkeys, for which we were able to generate reliable genotypes for all 13 loci, were calculated using the software SPAGeDi, v.1.2 [36] We calculated both the Queller & Goodnight [37] and Wang [38] estimators, as previous studies have showed that performance of relatedness depends mainly on the population relatedness composition [39].

### Data analyses and statistics

To test for similarities in cleaning techniques within groups and within matrilines we used linear mixed effect models as implemented in the lme4 package for R [40]. We modelled the number of occurrences of each behaviour as a function of sex and age class (juveniles or adults) as fixed effects and experiment, matriline and group as random effects. In order to test whether these effects differed from 0, we used the HPD-interval function of the lme4 package (see supplements Table 1A and 1B, for detailed results). This function creates highest posterior density (HPD) intervals for the parameters of a linear mixed effect model from Markov Chain Monte Carlo sampling of the fitted model. Non overlapping of the 99.9% HPD intervals with zero was taken as evidence against the null hypotheses of no effect of the variable at the 0.001 threshold R was also used for testing the correlation of similarity between matriline members and full sister adult females.

For the assessment of a potential genetic basis of different handling techniques we compared similarity indexes between mothers and their offspring with the similarity between full sisters. Spearman rank correlations over experimental sessions were calculated for the two most common behaviours, “no cleaning” and “hand rubbing”. We calculated one mean correlation coefficient for full sisters by taking the mean of the three values of groups that contained full sisters (2 full sisters in the Donga group, 3 full sisters in the Fishing Camp group and 4 full sisters in the Bay group). We then compared the means (Hand rubbing: −0.04; no cleaning: 0.091) with our similarity coefficients for mother-offspring pairs, where we calculated mean values for mothers that had several offspring to conduct Wilcoxon one sample tests (18 “hand rubbing” and 19 “no cleaning” mother-offspring data, the difference in sample size between the two techniques is due to missing data, with one matriline not using the “hand rubbing”), using SPSS 16.0.

To test whether individuals preferentially ate in the presence of matriline members we first excluded adult males from the data set and then calculated for each dyad of group members the amount of scans present together at the box divided by the mean of the amount of scan each one was present in total. In order to avoid dependencies in the data we calculated one mean value per group for the percentage of feeding events in the presence of matriline members and the percentage of feeding events in the presence of other group members for a matched pair comparison.

For the analysis on the development of individual handling techniques over the sessions we used techniques (the four cleaning techniques and ‘no cleaning’) used in session 15 as reference and compared the results with the results of sessions 2–14 and with session 16 (minimum 1 year later). We omitted session 1 because one group did not eat any fruits during the first session. Our similarity index scored the number of fruits that were handled in the same way in each pairwise comparison between sessions. As the handling of ten grapes was observed for each individual in each session, individual scores could take any decimal value between 0 and 1. Based on individual scores we first calculated a mean similarity score per group and based on these values a mean similarity score per session. We used these values for a Spearman rank correlation to test whether individual methods stabilised over the course of the experiment. The evaluation of the stability of methods used after a year is descriptive.

## Results

The five different handling techniques were already used during the first session where a total of 63 monkeys ate. Individuals used typically more than one technique already during the first session. The most common grape handling used during the first session were no cleaning (used by 78% of individuals) and the cleaning technique rubbing in hands (used by 51%). Less common were rubbing on substrate (ground, branches, stones, the plastic box, 25%), opening the grape with mouth and not eating the peel (25%). The fifth method, opening the grape with hands and eating the inside, was done by only one monkey at this stage (1%).

### Units of similarity in handling techniques

**Figure 1:**
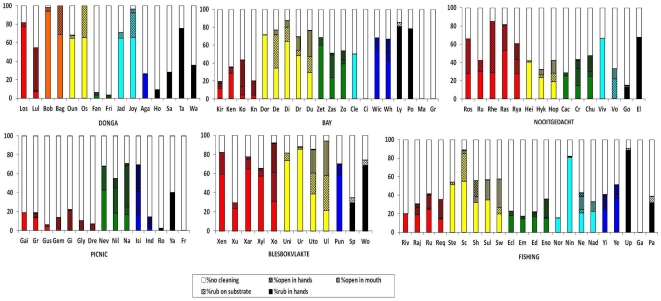
Percentage of cleaning techniques in the 6 groups. Each bar represents one individual, where three letter codes represent females and two letter codes represent males. The same colour bars are used to represent members of the same matrilines. Matrilines are ordered following the hierarchical structure (dominant on the left-subordinate on the right). Adult males are in black, again ordered following hierarchy.

A significant proportion of the variance was accounted for by matriline membership with respect to all five handling techniques (generalized linear mixed effects models: all five 99.9% highest posterior density (HPD) intervals did not overlap with 0, Fig. 1, table S1). The results remain robust for all sand removal techniques if the observations of ‘non cleaning’ are removed from the analysis, confirming that our method evaluated each technique independently (results not shown). For the opening grapes with mouth, we additionally found an age effect as this technique was more frequently used by juveniles than by adults (p-value <0.001, Fig. 1, table S1). In contrast, we never found significant effects of group identity or the sex of individuals (p-value >0.2, all HPD intervals at 80% overlapped with 0, Fig. 1; table S1). The results remained stable when we considered only females for our analyses (results not shown).

### Development and stability of foraging techniques

**Figure 2:**
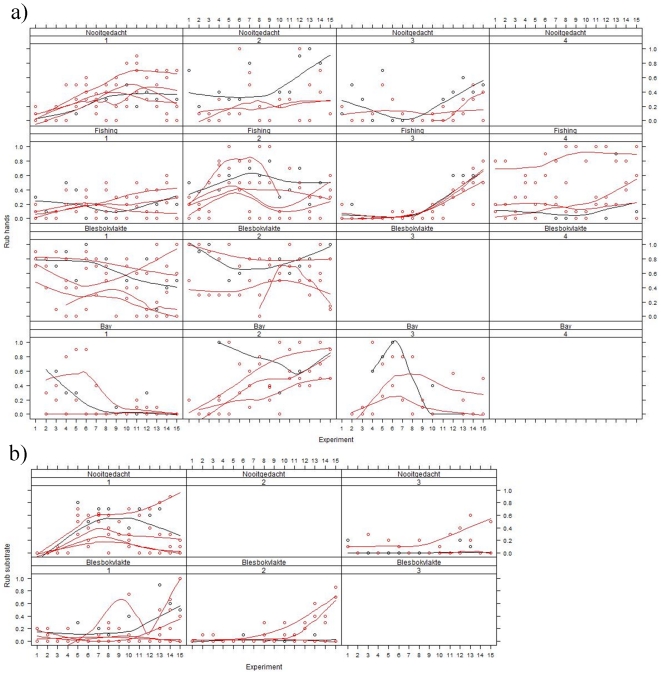
Examples of the development of the propagation of cleaning techniques within matrilines. 2a) rubbing grapes in hands; 2b) rubbing grapes on substrate. Only matrilines for which 2 individuals or more participated in at least 6 experiments are represented. On each panel, the mother female is represented in black, other individuals with red. The lines represent a running median smoother based on three consecutive data points (J. W. Tukey, Exploratory Data Analysis, Reading Massachusetts: Addison-Wesley, 1977). Above each panel the group name is given and the matriline's rank in the hierarchy.

**Figure 3:**
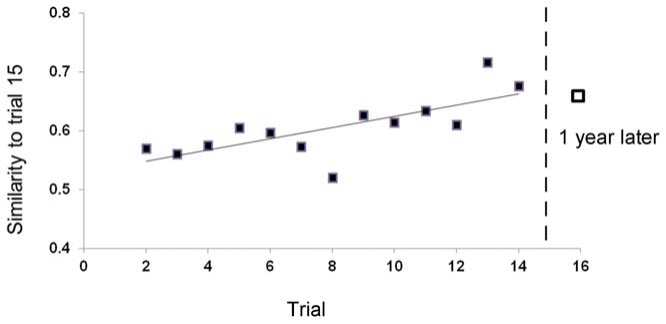
Similarity index between the individuals' handling technique in session 15 and all other sessions. Mean of mean group values for each session. Line shows linear regression. Session 16 was conducted at least one year later and hence represents an extra experiment, indicated by its separation from the other data points by a dashed line.

We found a session effect for all behaviours except of opening grapes with mouth, showing that individuals alter the relative frequency of techniques used across sessions while at the same time adapting their choice to the techniques used within their matriline (generalized linear mixed effects models: HPD intervals at 99.9% did not overlap with 0 for non cleaning, rubbing in hands and on substrate, and opening grapes with hands; p-value >0.1 for opening grapes with mouth, Fig. 2). Within individuals, the consistency of methods used stabilised across sessions: the similarity in methods between the final session 15 and previous sessions correlates positively with session number (Spearman rank correlation, n = 13, rs = 0.797, p = 0.001, Fig. 3). Apparently, the methods used at the end were pretty stable because the similarity to the methods used one year later was high (Fig. 3).

### Simultaneous foraging

**Figure 4:**
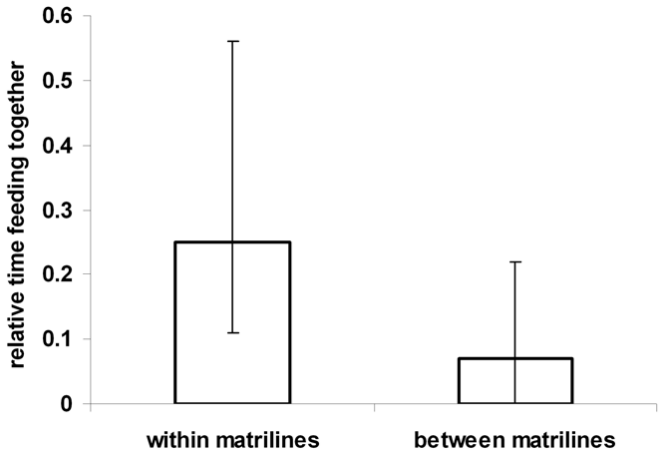
Time spent foraging with matriline members and with others. Median and interquartiles of the proportion of time persons spent foraging together with members of the same matriline and with all other group members.

Members of the same matriline were more likely to feed at the box with each other than with other group members (Wilcoxon signed ranks test, n = 6 means per group for matriline members and for non matriline members, Z = −2.201, p = 0.028, Fig. 4). We therefore investigated whether the similarities in handling techniques within matrilines described above could be explained with simultaneous foraging or whether similarities persisted also when matriline members ate separately. When we reran the analyses and considered only observations where individuals ate grapes in the absence of matriline members the matriline effect persisted in four of the five methods (generalized linear mixed effects models: HPD intervals at 99.9% did not overlap with 0 for non cleaning, rubbing in hands and on substrate, and opening grapes with hands; p-value >0.1 for opening grapes with mouth; table S2).

### Matriline effects versus genetic relatedness per se

**Figure 5:**
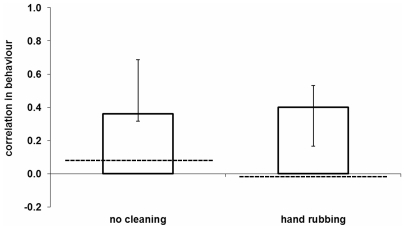
Similarity in grape handling between mothers and their offspring for the two most common techniques, no cleaning and rubbing in hands. Median and interquartiles of correlation coefficients for individual matrilines. Dashed lines: mean correlation coefficients between adult full sisters (0.091 for ‘no cleaning’; −0.04 for ‘hand rubbing’).

To test whether genetic relatedness per se could explain the matriline effects we first calculated the average correlation in behaviour between adult full sisters with respect to the two most commonly used methods, namely eating the grapes without cleaning and rubbing them with the hands. The average values were then compared with the correlation in behaviour for each adult female and her offspring. For both “no cleaning” and “rubbing in hands”, we found that adult females behaved more similarly to their offspring than full sisters to each other, despite the same level of relatedness (Wilcoxon sign ranked test (mean of 3 groups with full sister pairs vs 18 mother-offspring pairs), for no cleaning: N = 19, V = 156, p = 0.0124, for rubbing in hands: N = 18, V = 156, p-value = 0.001, Fig. 5).

## Discussion

Our study provides evidence that individuals choose to behave similarly to key peer members. Interestingly, the key unit for behavioural similarity in our experiment was the matriline and not the philopatric sex or even the whole group. The importance of matriline membership for social learning in primates has been reported before in a study on the diet and foraging skills of wild orangutans [24] as well as capuchins [23] and also as in various studies on Japanese macaques [25]–[27] but we had expected a group effect as well. The expectation was based on experimental evidence collected on the same study groups that dominant female models (but not dominant male models) caused social learning in non-matriline group members in an artificial fruit experiment [11]. The current results suggest that in vervet monkeys social learning from non-matriline females may be important in novel situations while behavioural similarity is restricted to matriline members in more daily life circumstances. Simultaneous actions are likely to be important for such behavioural matching, and our proximity results emphasize the likely importance of tolerance and proximity to enable opportunities to copy behaviours.

### Potential mechanisms of similarity

The matriline effects could in principle be explained with three different mechanisms. The similarity could be based on momentary conversion, on genetic effects or on social learning. If momentary conversion could explain the results we expected that the matriline effect would only be found during events in which matriline members co-fed and that the similarity disappears if we consider only observations where individuals ate in the absence of matriline members. However, this was not the case; similarities persisted when individuals fed in the absence of matriline members. With respect to the genetic hypothesis we note that two results contradict its predictions. First, individuals were flexible in their use of different methods and altered methods over time, converging with the methods used by other matriline members. Convergence implies social learning. In addition, matriline members used more similar methods than adult full sisters did. This result could potentially be explained with epigenetic effects but not with the presence/absence of specific alleles. In conclusion, it appears that social learning is the most parsimonious explanation for our results.

### Potential social learning mechanisms

While our experimental design does not allow strong conclusions about the potential underlying social learning mechanisms there are a few interesting hypotheses for future studies. First, we note that all handling techniques were present during the first session. Thus, it appears that they form part of the vervets' natural behavioural repertoire, making innovation followed by production imitation (sensu [41]) an unlikely scenario. As it stands, evidence for production imitation is currently restricted to laboratory studies [3], [6], [42], [43], though these claims and the definitions have been challenged [41]. In contrast, the data indicate that the vervets used social learning, at least response facilitation, i. e. the presence of a demonstrator performing an act increases the probability of an animal which sees it doing the same, or maybe also contextual imitation, i. e. the imitation of known behaviours in new contexts [41]. Foraging simultaneously with other matriline members they could observe the techniques used by other members and then converge on these techniques and keeping them even in the absence of matriline members. This explanation appears to fit in particular the opening of grapes with either the mouth or the hands as simpler social learning mechanisms like ‘stimulus enhancement’ or ‘local enhancement’ [41] do not seem to explain these techniques. In fact, a classic experiment demonstrating imitation learning in marmoset monkeys took advantage of models using teeth or hands to open a filmbox [44]. Contextual imitation has also been demonstrated in a field experiment on egg handling in mongooses [45]. Thus, social learning through contextual imitation may be quite widespread in wild mammals. On the other hand, rubbing the grapes on a substrate instead of with both hands might be due to local enhancement. Observers are attracted to a location and then perform an individually chosen behaviour. Clearly, more explicit experiments aiming at testing the social learning mechanisms used by vervet monkeys are needed.

### Social learning leading to traditions?

Individual feeding habits during the last two sessions were about as similar to each other as to the extra session after at least one year of pause. This result suggests that individual feeding techniques and similarities within matrilines stabilised. Stabilisation of behaviour based on social learning eventually leads to the formation of traditions. A future study could take our results one step further and investigate whether a new generation of monkeys will adopt similar techniques as matriline members. Such traditions would be expected on the matrilineal level rather than the group level. This is in sharp contrast to some previous studies that proposed the existence of traditions on the population level based on differences in behaviour that seem to lack an ecological explanation [13]–[15] though only the study on orang-utans provided population level observations rather than observations on one to few groups per population. On the other hand, wild capuchin monkeys provide some evidence for arbitrary traditions within populations. In this species, social conventions have been reported as inserting fingers into the mouth, nostrils and even eyes of group members and a variety of ‘games’ in which small objects such as hairs are put in one monkey's mouth and extracted by another [46]. Also arbitrary variation in food processing has been reported in capuchins, where the seeds of Luehea fruits can be extracted in two alternative ways of similar efficiency, and while juveniles eventually try both methods during their development, at least young females converge on the technique their mothers used [23].

### Social learning rules

Our results have important implications for the theoretical framework of social learning rules. Depending on the circumstances and/or the identity of potential models, individuals may decide to use social information [16], [18], [20], [22], [47], [48]. In fish it has been shown that individuals can compare their own foraging success with the success of potential models in order to decide whether to copy decisions of a model [49]–[50]. For primates, which live in stable social groups, it has been proposed that specific individuals may act as role models: the mother or a dominant individual [18] or maybe more specifically a member of the philopatric sex [11]. According to the model by Yeaman et al. [22], a social learning rule that details that the mother and (high ranking) members of the philopatric sex are suitable models, would lead to similarities on the group level in species with male migration. Similarity of behaviours on the group level may be further promoted by conformity: individuals prefer to behave as the majority of individuals in a group [16] or perhaps specifically copy the behaviour they observe most often [48].

In contrast to the concepts presented above, our results suggest that in vervets the mother as role model may be of much higher importance than any other group member, while evidence for conformity is absent. Note, however, that our experimental design does not allow us to evaluate who learned from whom. In principle, we cannot exclude that mothers learned from their offspring. Thus, the precise social learning rules of vervet monkeys need further experimentation, ideally not only in a foraging situation but also in other contexts. In vocalisation studies, matrilineal song patterns were shown in killer whales [51], though later results also demonstrated the importance of horizontal transmission between adults [52]. In a group living songbird, the stripe-backed wren (*Campylorhynchus nuchalis*) sex-specific social learning apparently results in call traditions following separately patrilines and matrilines [53].

In conclusion, we hope that our results will inspire theoreticians interested at exploring the adaptive value of different social learning rules in different contexts. Our current results fit the hypothesis that behavioural conformity on the group level is only prominent in humans and chimpanzees [54]. The restriction of traditions to matriline membership rather than to the group level may hinder the development of culture in monkeys relative to apes or humans. We believe that our experimental design would likely yield variable handling behaviour in many species, allowing the use of a comparative approach to explore the units of social learning and behavioural similarity across species.

## Supporting Information

Table S1
**99.9% Highest Posterior Density intervals from linear mixed effects models.** Model is
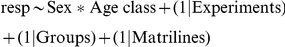
where resp is one of No cleaning, Rub in hands, Rub on substrate, Open in mouths or Open in hands. Observed effect and 99.9% highest posterior density intervals for the different explanatory variables in the linear mixed effect models. The intercept is the predicted value for juvenile females, line Sex gives what needs to be added to the intercept to obtain the predicted value for males, line Age class gives what should be added to the intercept to obtain the predicted value for adults. For random effects, the standard deviation of the effect is given, and the HPD represents the proportion of standard deviation relative to the residual standard deviation explained by the effect. Intervals that do not include 0 are significant at the 0.001 level and are shown in bold.(DOCX)

Table S2 **Sub-sample of table S1 for matrilineal members feeding without their matrilines.** 99.9% Highest Posterior Density intervals from linear mixed effects models.
(DOCX)
