# Supplementary material for: Similarity in Food Cleaning Techniques within Matrilines in Wild Vervet Monkeys
Source: PLoS One. 2012 Apr 25;7(4):e35694. doi: 10.1371/journal.pone.0035694 (PMC3338447; doi:10.1371/journal.pone.0035694)
Supplement: Table S1 — 99.9% Highest Posterior Density intervals from linear mixed effects models. Model iswhere resp is one of No cleaning, Rub in hands, Rub on substrate, Open in mouths or Open in hands. Observed effect and 99.9% highest posterior density intervals for the different explanatory variables in the linear mixed effect models. The intercept is the predicted value for juvenile females, line Sex gives what needs to be added to the intercept to obtain the predicted value for males, line Age class gives what should be added to the intercept to obtain the predicted value for adults. For random effects, the standard deviation of the effect is given, and the HPD represents the proportion of standard deviation relative to the residual standard deviation explained by the effect. Intervals that do not include 0 are significant at the 0.001 level and are shown in bold. (DOCX) [file pone.0035694.s001.docx]

**Table S1.**

|  | No cleaning | Rub in hands | Rub on substrate | open in mouth | open in hands |
| --- | --- | --- | --- | --- | --- |
|  |  |  | Fixed effects |  |  |
| Intercept | [1.42, 7.15] | [-0.15, 5.32] | [0.03, 1.64] | [0.54, 1.36] | [-0.34, 0.34] |
| Sex | [-0.51, 1.19] | [-0.96, 0.53] | [-0.55, 0.29] | [-0.30, 0.31] | **[ 0.02, 0.30]** |
| Age class | [-0.10, 1.55] | **[ 0.62, 2.10]** | [-0.61, 0.18] | **[-1.22,- 0.63]** | [0.00, 0.26] |
| Sex : Age Class | [-1.54, 3.01] | [-1.90, 2.09] | [-1.15, 0.45] | [-0.58, 0.49] | [-0.57, 0.03] |
|  |  |  | Random effects |  |  |
| Matrilines | **2.47[ 0.45, 0.85]** | **2.27[ 0.45, 0.89]** | **0.54[ 0.21, 0.55]** | **0.28[ 0.12, 0.41]** | **0.26[ 0.32, 0.68]** |
| Groups | 0.79[0.00, 1.66] | 0.70[0.00, 1.34] | 0.10[0.00, 1.16] | 0.00[0.00, 0.51] | 0.06[0.00, 1.13] |
| Experiments | **0.63[ 0.09, 0.54]** | **0.57[ 0.09, 0.53]** | 0.00[0.00, 0.22] | 0.00[0.00, 0.19] | 0.02[0.00, 0.23] |
| Residuals | 2.80[2.65, 3.04] | 2.58[2.45, 2.80] | 1.43[1.34, 1.53] | 1.10[1.03, 1.18] | 0.47[0.44, 0.50] |
