# Supplementary material for: Similarity in Food Cleaning Techniques within Matrilines in Wild Vervet Monkeys
Source: PLoS One. 2012 Apr 25;7(4):e35694. doi: 10.1371/journal.pone.0035694 (PMC3338447; doi:10.1371/journal.pone.0035694)
Supplement: Table S2 — Sub-sample of table S1 for matrilineal members feeding without their matrilines. 99.9% Highest Posterior Density intervals from linear mixed effects models. (DOCX) [file pone.0035694.s002.docx]

**Table S2.**

|  | No cleaning | Rub in hands | Rub on substrate | open in mouth | open in hands |
| --- | --- | --- | --- | --- | --- |
|  |  |  | Random effects |  |  |
| Matrilines | **[0.29, 0.74]** | **[0.26, 0.78]** | **[0.13; 0.51]** | **[0.13; 0.47]** | **[0.13; 0.51]** |
| Groups | [0.00, 1.75] | [0.00, 1.16] | [0.00, 0.77] | [0.00, 0.50] | [0.00, 0.93] |
| Experiments | **[0.11, 0.47]** | **[0.09, 0.38]** | **[0.07; 0.34]** | [0.00; 0.18] | **[0.05; 0.30]** |
